# Degradation and Stabilization of Inverted Perovskite Solar Cells

**DOI:** 10.3390/nano16191223

**Published:** 2026-09-28

**Authors:** Runquan Qi, Anqi Wang, Guiran Gao, Menglin Tian, Zhibo Hou, Ke Guo, Guangbao Wu

**Affiliations:** 1 State Key Laboratory of Flexible Electronics (LoFE) & Institute of Advanced Materials (IAM), Nanjing University of Posts & Telecommunications, 9 Wenyuan Road, Nanjing 210023, China; b23060208@njupt.edu.cn (R.Q.); 2025060506@njupt.edu.cn (G.G.); b24060302@njupt.edu.cn (M.T.); b24060427@njupt.edu.cn (Z.H.); 2 School of Biological & Chemical Engineering, Qingdao Technical College, No. 369 Qian Tang Road, Huangdao District, Qingdao 266555, China; 18763728507@163.com

**Keywords:** perovskite solar cells, stability, degradation mechanisms

## Abstract

Inverted small-area perovskite solar cells fabricated in the laboratory have achieved a power conversion efficiency of 28%. Nevertheless, stability remains the key bottleneck. This review systematically analyses external stressors moisture, heat, light, oxygen, electric field, intrinsic ion migration and interfacial defects, alongside stabilization strategies including composition, passivation, 2D/3D heterojunctions, facets engineering, and encapsulation. Synergistic stressors produce nonlinear degradation; effective strategies must tackle multiple pathways simultaneously without impairing charge transport.

## 1. Introduction

Metal-halide perovskite solar cells (PSCs) have developed rapidly since their first photovoltaic demonstration in 2009. Their strong absorption, tunable bandgap, long carrier diffusion length, defect-tolerant electronic structure, and compatibility with low-temperature solution or vapor processing make them attractive for both rigid and flexible photovoltaics [1]. According to the 2026 NREL efficiency tables, the certified efficiency of single-junction PSCs had risen from 3.8% to about 28%, placing this technology in the high-efficiency range of established thin-film photovoltaics [2]. 

PSCs are commonly fabricated in the conventional n-i-p or inverted p-i-n configuration. A typical inverted device follows the sequence transparent conductive oxide/hole-transport layer/perovskite/electron-transport layer/metal electrode. This architecture supports low-temperature processing and is compatible with thin transport layers and flexible substrates [3]. Recent studies have further shown that the control of buried and top interfaces can suppress non-radiative recombination and reduce the performance gap between inverted and conventional devices [4,5,6]. Nevertheless, efficiency alone is not sufficient to establish technological viability. The perovskite absorber is an ionic, structurally soft semiconductor whose composition and defect population can change under moisture, heat, illumination, oxygen, and electrical bias [7,8,9,10,11]. These stressors may drive hydration, phase transformation, volatile-product loss, redox reactions, defect redistribution, and ion migration. The resulting changes are often coupled in that a defect induced by one stressor can accelerate the response to another stressor [12,13,14]. 

This review focuses on the stability of inverted PSCs, with the perovskite layer as the central object of analysis. Section 2 introduces the main functional layers and their roles without extending the discussion to the environmental aging of non-perovskite layers. Section 3 and Section 4 then examine external and operational stressors acting on the perovskite, together with intrinsic ionic and interfacial degradation pathways. Section 5 evaluates material, interface, structural, mechanical, and encapsulation strategies, and Section 6 discusses the remaining challenges for reliable lifetime prediction and scale-up.

## 2. Functional Design of Contacts and Interfaces in Inverted Perovskite Solar Cells

The performance of an inverted perovskite solar cell is governed not by the isolated properties of individual layers, but by the chemical and electronic coupling between them (Figure 1) [3]. The bottom contact must combine selective hole extraction with a surface that supports uniform perovskite nucleation. The absorber must provide efficient photocarrier generation while tolerating ionic rearrangement and interfacial reactions. The upper contact must extract electrons without damaging the perovskite or enabling metal and halide diffusion. These requirements are intrinsically coupled, making contact chemistry and interfacial structure as important as bulk transport properties.

### 2.1. Hole-Transport Layer

The central challenge for the hole-selective contact is to provide efficient hole extraction without compromising perovskite nucleation or chemical stability [15]. Designing the HTL is therefore a balancing act: hole extraction, wetting and nucleation, chemical inertness, ion blocking, and thermal expansion matching must all be balanced simultaneously [16]. 

Organic hole-transport layers such as PEDOT:PSS and PTAA are attractive because they can be processed at low temperature and form optically transparent contacts. Their surface chemistry, however, creates different limitations. The acidic and highly polar character of PEDOT:PSS can promote undesirable reactions with the perovskite or the transparent electrode, whereas the hydrophobic surface of PTAA can lead to incomplete wetting and nonuniform nucleation. These limitations have motivated the use of surface modifiers and ultrathin interlayers that regulate both interfacial energetics and precursor wetting [17,18]. 

Inorganic NiO_x_ offers higher thermal robustness, but its performance is controlled by defect chemistry rather than by nominal composition alone. The Ni^2+^/Ni^3+^ ratio, surface hydroxyls, residual precursor species, and the energetic offset with the perovskite all influence conductivity, recombination, and chemical reactivity. Precursor chemistry, pH control, doping, and molecular anchoring can therefore improve NiO_x_ contacts by simultaneously tuning the work function and reducing interfacial defects [19,20,21]. This comparison highlights a broader design principle: the most effective hole-selective contact is not necessarily the one with the highest intrinsic conductivity, but the one that provides the most favorable combination of energy alignment, surface reactivity, and film-forming compatibility.

Self-assembled monolayers offer a more molecularly defined approach. Their anchoring groups bind to the conductive oxide, while the conjugated backbone and terminal group regulate charge extraction and interaction with the perovskite precursor [22,23,24]. In this case, molecular packing, surface coverage, dipole orientation, and resistance to solvent-induced disorder become critical. SAMs therefore shift the design problem from bulk transport to nanoscale control of the buried interface.

### 2.2. Perovskite Absorber Layer

The perovskite absorber is not merely the light-harvesting layer; it is also the chemically active component that responds to heat, light, bias, and contact-induced reactions. Composition determines the bandgap and phase stability, but it also affects defect formation, ionic mobility, and interfacial reactivity. MAPbI_3_ crystallizes readily but suffers from the volatility and thermal instability of methylammonium. FAPbI_3_ provides a narrower bandgap and is widely used in high-efficiency devices, although its photoactive α-phase is metastable near room temperature [25,26]. Mixed-cation compositions containing FA, Cs, and limited amounts of MA are therefore used to balance bandgap, crystallization behavior, and phase stability rather than to optimize any single parameter [27].

Low-dimensional surface phases and lead-free tin perovskites should be considered as distinct strategies rather than as equivalent routes toward a “more stable” absorber. A thin 2D or quasi-2D phase can reduce surface defect density and suppress ion exchange, but excessive thickness, poor orientation, or deep penetration can impede out-of-plane charge transport [28,29,30]. The relevant design variables are therefore the location, continuity, orientation, and chemical persistence of the low-dimensional phase. In contrast, tin-based absorbers address lead reduction and bandgap tuning but introduce strong sensitivity to Sn^3+^ oxidation and self-doping [31]. Their limitations arise from a different chemical mechanism and should not be conflated with those of 2D/3D interfaces.

### 2.3. Electron Transport and Buffer Layers

The upper contact must provide selective electron extraction while protecting the perovskite from the deposition process and subsequent metal diffusion. Fullerene derivatives remain widely used because they offer favorable electron affinity and conformal coverage of the perovskite surface. Their practical limitations are associated less with light absorption than with film continuity, molecular aggregation, energetic disorder, and resistance to solvent or thermal treatment. Cross-linkable fullerene networks, controlled PCBM morphology, and interfacial doping are consequently designed to improve contact uniformity and mechanical integrity [32,33]. These strategies address film continuity and contact uniformity rather than changing the intrinsic absorption properties of the perovskite. 

Inorganic SnO_x_ can provide compact and optically transparent electron-selective layers, but the deposition chemistry must be compatible with the chemically soft perovskite surface. Composite electron contacts can further combine passivation, work function adjustment, and improved extraction [34]. Buffer layers such as bathocuproine are used to decouple the fullerene from the metal electrode; however, excessive thickness introduces transport resistance and can produce charge accumulation or an S-shaped current–voltage response [35]. 

### 2.4. Top Electrode

The metal electrode should likewise be regarded as a chemically active part of the device rather than as an inert external conductor. Ag and Cu offer attractive conductivity and processing advantages, but they can react with mobile halides or decomposition products. Diffusion barriers and chemical inhibitors are therefore needed to stabilize the electrode without altering the absorber itself [36]. 

### 2.5. Interfaces and Functional Interlayers

The buried HTL/perovskite and perovskite/ETL interfaces determine nucleation, local strain, defect populations, band bending, carrier selectivity, and ion transport [37]. Their behavior cannot be inferred from the properties of the adjoining bulk layers alone. Molecular modifiers, ultrathin interlayers, and low-dimensional surface phases are effective only when they simultaneously improve electronic selectivity, suppress interfacial reactions, and preserve continuous charge transport.

## 3. Effects of External and Operational Stressors on the Perovskite Layer

### 3.1. Moisture

Moisture degradation begins with adsorption and transport rather than with an instantaneous bulk reaction. Water molecules preferentially adsorb at iodide vacancies, undercoordinated Pb^2+^ sites, grain boundaries, pinholes, and surfaces with incomplete organic-cation coverage. Hydrogen bonding to halides and coordination to Pb^2+^ locally weaken the Pb-I bonds, thereby lowering the barrier for water ingress into the lattice (Figure 2). The rate-determining step can therefore shift from surface adsorption at low humidity to grain-boundary diffusion and dissolution at higher water activity. Under ambient humidity, the perovskite first forms hexagonal polytype intermediate phases and then decomposes into various products; to a certain extent, increasing the A-site Cs content enhances stability, while increasing the X-site Br content accelerates degradation [38]. 

For MAPbI_3_, exposure to moisture initiates degradation through a stepwise pathway. Initially, water molecules intercalate into the perovskite lattice, forming an unstable and reversible hydrate intermediate (MAPbI_3_·H_2_O, Equation (1)). This intermediate phase subsequently decomposes into PbI_2_ and the organic component MAI (Equation (2)). Under humid and ambient air conditions, MAI further undergoes degradation, yielding volatile HI and MA gases (Equation (3)). The released HI is unstable: in the presence of oxygen, it is oxidized to I_2_ and H_2_O (Equation (4)), while under ultraviolet irradiation, HI can also decompose into H_2_ and I_2_ (Equation (5)) [39]. The evolution of these volatile species ultimately results in irreversible degradation of the material. The overall reaction pathway can therefore be summarized as follows: [40]

(1) $$CH_{3}NH_{3}PbI_{3}+H_{2}O↔\;{CH_{3}NH_{3}\mathrm{PbI}}_{3}\cdot H_{2}O$$

(2) $${CH_{3}NH_{3}\mathrm{PbI}}_{3}\cdot H_{2}O\to {\mathrm{PbI}}_{2}+CH_{3}NH_{3}I+H_{2}O$$

(3) $$CH_{3}NH_{3}I\overset{H_{2}O}{↔}HI+CH_{3}{NH}_{2}$$

(4) $$4HI+O_{2}\;\to 2I_{2}↑+\;2H_{2}O$$

(5) $$2HI\to H_{2}↑+\;I_{2}↑$$

The electrical loss precedes complete conversion to PbI_2_. Lattice expansion, partial dissolution, and repeated hydrate formation generate vacancies, distorted Pb–I–Pb linkages, and discontinuous grain contacts. These changes increase energetic disorder and non-radiative recombination, reduce carrier mobility, and create additional sites for water adsorption. Moisture degradation is therefore self-accelerating: the initial reaction creates defects and open diffusion paths that promote the next reaction step [41]. 

For FAPbI_3_, moisture not only drives the transformation from the photoactive α-phase to the non-photoactive δ-phase, but also cooperates with bromide ions to form hydrated byproducts. Studies have shown that under high-humidity conditions, α-FAPbI_3_ gradually decomposes into the δ-phase, PbI_2_, and FAI (Equation (6)). In the presence of water, FAI further reacts with Br^−^ to yield a hydrated salt, FABr·H_2_O (Equation (7)). This sequence of reactions leads to progressive destruction of the perovskite lattice, which is reflected in XRD measurements by the emergence of δ-phase peaks along with a new diffraction signal at 13.04° (Equation (8)). Taken together, these results indicate that moisture not only directly triggers phase transformation, but also accelerates performance degradation by facilitating the formation of hydrated byproducts [42].

(6) $$\alpha -perovskite\overset{H_{2}O}{\to }\delta -perovskite+FAI+{\mathrm{PbI}}_{2}$$

(7) $${FA}^{+}+{Br}^{-}\overset{H_{2}O}{\to }\mathrm{FABr}\cdot H_{2}O$$

(8) $$FAI+{\mathrm{PbI}}_{2}\overset{H_{2}O}{↔}\delta -perovskite$$

### 3.2. Heat

Heat acts through four coupled pathways: reversible structural phase transitions, irreversible chemical decomposition, thermally activated defect and ion motion, and thermomechanical stress. These pathways have different activation energies and time scales. The loss of volatile A-site or halide-containing products produces a permanent stoichiometric change (Figure 3).

MAPbI_3_ undergoes a tetragonal-to-cubic transition as the temperature increases. Increased rotational freedom of MA^+^ and reduced octahedral tilting make the average lattice more symmetric, changing Pb–I–Pb angles, dielectric response, and transport. The transition can redistribute strain and defects, but it is not by itself an irreversible failure. Chemical degradation begins when thermal energy is sufficient to break the equilibrium between MAPbI_3_ and its volatile components [43,44]. 

A common thermally induced pathway for MAPbI_3_ is decomposition into PbI_2_ and MAI (Equation (9)), followed by dissociation and loss of methylamine and HI (Equation (10)). Because CH_3_NH_2_ and HI can escape through grain boundaries and interfaces, an initially reversible equilibrium becomes irreversible in device. The resulting A-site and iodide deficiencies leave behind undercoordinated Pb species, iodide vacancies, and PbI_2_ rich regions.

(9) $$CH_{3}NH_{3}PbI_{3}↔PbI_{2}+CH_{3}NH_{3}I$$

(10) $$CH_{3}NH_{3}I↔CH_{3}{NH}_{2}+HI↑$$

For FAPbI_3_, temperature has a dual role. The cubic α phase gains configurational and vibrational entropy at elevated temperature, whereas the yellow δ phase is favored near ambient conditions when the α phase is not kinetically stabilized. Cooling, defects, residual strain, and heterogeneous nucleation sites can lower the barrier for α-to-δ conversion. Thus, high-temperature retention of the black phase does not guarantee room-temperature phase stability after cooling. FA-based perovskites also undergo irreversible chemical decomposition at sufficiently high temperature. Coupled thermogravimetry–mass spectrometry has detected hydrogen cyanide and sym-triazine-related products during degradation of formamidinium lead halides. This pathway involves chemical transformation of the FA component rather than simple evaporation of intact FAI, demonstrating that the higher thermal robustness of FA-rich absorbers is relative rather than absolute [45,46]. 

Temperature accelerates ionic and defect kinetics according to activated transport. Higher thermal energy increases the concentration and mobility of halide vacancies and interstitials, enables redistribution of charged defects, and shortens the time required for ions to reach grain boundaries and interfaces. The resulting ionic space charge changes local electric fields and recombination rates; under electrical operation, self-heating and ionic motion can therefore reinforce transient hysteresis and long-term degradation [47]. 

Thermomechanical damage arises because the perovskite, substrate, transport layers, and electrode have different coefficients of thermal expansion and elastic moduli. Heating and cooling constrain the absorber biaxially, producing tensile or compressive strain. Repeated cycles concentrate stress at grain boundaries and weak interfaces, where cohesive cracking, interfacial delamination, and void formation can occur. These defects then expose new surfaces, accelerate volatile-product escape, and provide fast paths for moisture and oxygen. Heat also changes the rates of other chemical pathways. It accelerates oxygen diffusion and reaction at iodide-related defects, increases water transport and hydration kinetics, and raises ion mobility under illumination or bias. Consequently, damp heat, light soaking, and electrically loaded thermal tests may activate mechanisms that are absent in a dry, dark storage test [7]. 

### 3.3. Light

Illumination moves the perovskite away from its near-equilibrium ionic-semiconductor state and into a carrier-rich, non-equilibrium regime. Photogenerated electrons and holes shift the quasi-Fermi levels, redistribute defect charge states, generate local electric fields, and couple to the soft lattice. Light-induced instability is therefore not limited to direct photon-induced bond cleavage; it is often mediated by trapped carriers, redox reactions, and enhanced ionic motion (Figure 4) [48].

In mixed-halide perovskites, this non-equilibrium carrier accumulation promotes halide-ion migration and the formation of iodine-rich and bromine-rich microdomains. Importantly, this photoinduced halide segregation is generally reversible, with the material recovering after dark storage [49]. It is therefore better described as a carrier-mediated, reversible ionic rearrangement and should be distinguished from irreversible photodegradation involving permanent compositional changes, volatile-species loss, Pb^0^ formation, or structural decomposition [50,51]. In devices, Voc loss, hysteresis, and light-soaking effects do not necessarily prove that the material has decomposed; they may instead reflect the reversible ion electron dynamics [52].

Metallic Pb^0^ is one important photochemical product. Holes can oxidize iodide to neutral iodine species (Equation (11)), whereas trapped electrons can reduce Pb^2+^ to Pb^0^ after local iodide loss (Equation (12)). Neutral iodine atoms can combine to form I_2_ or react with I^−^ to form polyiodides (Equations (13) and (14)). Escape or interfacial capture of these iodine species leaves an iodide-deficient, Pb-rich region, while Pb^0^ introduces deep states and can catalyze further decomposition [53].

(11) $$I^{-}+h^{+}\to I^{0}$$

(12) $$Pb^{2+}+2e^{-}\to Pb^{0}$$

(13) $$2I^{0}\to I_{2}$$

(14) $$I_{2}+I^{-}⇌I_{3}^{-}$$

High-energy violet or ultraviolet photons can activate additional pathways by generating carriers near surfaces and buried interfaces, weakening molecular anchoring, and increasing iodine-related defect formation. Photodegradation has also been observed under oxygen-free conditions, showing that oxygen is not required for all light-induced reactions. Light usually acts together with heat and the electric field. Non-radiative recombination and resistive losses raise the local temperature, while the built-in field directs the defects whose concentration and mobility have been increased by illumination [54,55].

### 3.4. Oxygen

Oxygen degradation is strongly light-dependent. In the dark, molecular O_2_ may adsorb weakly or shift equilibria by consuming HI and other reduced decomposition products; the rate depends on the halide composition and defect density. Under illumination, photogenerated electrons provide a direct reduction pathway, so oxygen becomes much more reactive. Iodide vacancies are key adsorption and electron-transfer sites. O_2_ diffuses rapidly through defect-rich films and can accept an electron from the perovskite conduction band or a trapped state to form superoxide (Equation (15)). Because vacancy-rich regions also support faster ion transport, oxygen adsorption and ionic redistribution reinforce each other: migration creates reactive sites, and reaction at those sites changes local stoichiometry and creates additional defects (Figure 5).

(15) $$O_{2}+e^{-}\to O_{2}^{-}$$

When perovskite materials are simultaneously exposed to oxygen and light, the degradation is substantially faster. The light-accelerated degradation is attributed to the possible formation of superoxide ions under oxygen-containing conditions:

(16) $$CH_{3}NH_{3}PbI_{3}+light\to CH_{3}NH_{3}PbI_{3}^{-}$$

(17) $$CH_{3}NH_{3}PbI_{3}^{-}+O_{2}\to CH_{3}NH_{3}PbI_{3}+O_{2}^{-}$$

(18) $$4CH_{3}NH_{3}PbI_{3}+O_{2}^{-}\to 4CH_{3}NH_{2}+{4PbI}_{2}+2I_{2}+{2H}_{2}O$$

Here, MAPbI_3_^−^ denotes the perovskite in the excited state after photon absorption. The critical step in the formation of O_2_^−^ is the electron transfer from the conduction band of MAPbI_3_ (Equations (16) and (17)). The resulting O_2_^−^ is extremely reactive and can rapidly deprotonate the organic cation in the perovskite by cleaving the N–H bond, producing CH_3_NH_2_ (Equation (18)). The subsequent volatilization of CH_3_NH_2_ leads to irreversible material decomposition; once initiated, the degradation propagates quickly, causing deterioration of the perovskite solar cell [56]. O^2^-and photogenerated holes cooperatively oxidize iodide ions, yielding elemental iodine, neutral iodine interstitial atoms, and additional iodine vacancies. This leads to iodine loss, halide phase separation, and precipitation of metallic lead Pb^0^. In turn, the newly formed iodine vacancies further promote oxygen adsorption and electron transfer, establishing a self-amplifying destructive cycle that ultimately causes irreversible deterioration in device performance [57]. Recently, it has been proposed that if MAPbI_3_ absorbs photons of sufficiently high energy, photodegradation can occur even in the absence of molecular oxygen [58].

### 3.5. Electric Field and Electrical Bias

The built-in electric field in a perovskite solar cell helps electrons and holes move toward different contacts. However, carrier transport is not controlled by the electric field alone. Carrier diffusion and the selectivity of the transport layers are also important. Changes in the electric field can therefore affect band bending, charge extraction, carrier accumulation, and interfacial recombination (Figure 6). 

Perovskites conduct both electronic carriers and ions. The electric field not only drives electrons and holes, but also acts on charged defects. The flux of a mobile ionic defect can be described by

J_i_ = −D_i_∇c_i_ + z_i_μ_i_c_i_E

where D_i_ is the diffusion coefficient, μ_i_ the mobility, z_i_ the defect charge, and E the electric field. The first term describes diffusion caused by a concentration gradient, while the second term describes ion drift under an electric field.

Halide vacancies and interstitials usually migrate more easily than A-site cations or Pb-related defects. However, the main mobile species depends on the perovskite composition, defect concentration, temperature, microstructure, and operating time. Grain boundaries and interfaces can provide faster migration paths. Under continuous bias, positively and negatively charged defects move toward opposite interfaces. Their accumulation changes the internal electric field distribution and may partly screen the built-in field. This process changes band bending and carrier-extraction barriers, and can cause time-dependent changes in current, voltage, and capacitance. Ion redistribution can also contribute to current–voltage hysteresis, although trapping and interfacial recombination may also play a role [59].

At the early stage, ion migration mainly changes device performance through electrostatic effects. Field screening and changes in band bending can reduce carrier extraction, lower the fill factor, and increase carrier accumulation at interfaces. This promotes non-radiative recombination. Some of these changes may recover after the bias is removed because the ions can move back toward their original positions. Under long-term bias, ion redistribution may lead to irreversible chemical reactions. Halide species accumulated near metal electrodes can react with the metal and form metal halides, such as AgI. Local loss of iodide or A-site species can also create undercoordinated Pb sites and additional defects. Once mobile ions are trapped, electrode materials are consumed or volatile products are lost; the device cannot fully recover after the bias is removed. Reverse bias is more damaging because it can create a large local electric field, especially in partially shaded modules. Ions and carriers may accumulate at pinholes, local shunts, or weak interfaces. This can promote redox reactions, electrode corrosion, local heating, and electrical breakdown. Therefore, reverse-bias stability depends not only on ion migration in the perovskite, but also on transport layer coverage, interface quality, electrode stability, and device structure [11]. 

### 3.6. Coupled Degradation Network

The power conversion efficiency of perovskite solar cells is approaching that of crystalline silicon cells, yet stability remains the core bottleneck for their commercialization. The five environmental stressors—moisture, heat, light, oxygen, and the electric field—each degrade the perovskite lattice through distinct pathways. More critically, the synergistic coupling effects among these factors often produce destructive forces far exceeding the sum of individual contributions. Light invariably generates heat; this photo-thermal synergy drastically accelerates the detachment of organic cations and the oxidation of halides. When moisture and oxygen coexist, oxidative hydrolysis proceeds much faster than under dry conditions and penetrates deep into the grain interiors. In the simultaneous presence of light and water, superoxide radicals and hydroxyl radicals are generated on the perovskite surface, synergistically attacking the lattice framework and causing cleavage of lead–halide bonds and leaching of lead ions. The combined action of electric field and heat exacerbates ion accumulation at the electrodes and metal electrode corrosion, even inducing the formation of metal halides. This interwoven synergistic degradation network causes a nonlinear acceleration of lifetime decay under real-world operating conditions, posing the true challenge for perovskite cells transitioning from the laboratory to outdoor applications.

## 4. Intrinsic and Interfacial Degradation Pathways

### 4.1. Ion Migration

Beyond external stressors such as moisture, heat, light, electric fields, and oxygen, ion migration is widely recognized as a fundamental source of intrinsic instability in perovskite solar cells. In particular, iodide ions can migrate under very low electric fields, setting off a cascade of degradation processes. Migration generates and accumulates iodine vacancies and interstitial iodides, which introduce electronic defects. Simultaneously, escaped I^−^ can recombine to form I_2_, which under illumination readily converts into highly reactive iodine radicals [6]. These radicals dehydrogenate organic cations, triggering self-accelerating decomposition and structural collapse. Moreover, mobile I^−^ can diffuse toward metal electrodes, causing corrosion and irreversible device failure. As a result, perovskite solar cells often degrade within minutes under reverse bias, far below the reliability standards required for commercial photovoltaics [60].

### 4.2. Interfacial Chemical Reactions

The perovskite interfaces are chemically active because undercoordinated Pb, halide vacancies, organic cations, residual solvents, and mobile iodine species are concentrated near the film surface. Acids, bases, redox-active oxides, and reactive metals can shift local equilibria or catalyze decomposition. The relevant question is therefore not whether an interface is inert in isolation, but whether it remains compatible with the perovskite under carrier injection and ion accumulation.

Metal contacts provide a clear example. Iodide or iodine-containing species reaching Cu or Ag can form poorly conducting corrosion products and alter electrode morphology [61]. Chemical inhibitors and coordination layers can reduce these reactions by changing the metal surface chemistry and limiting bidirectional diffusion. Transport-layer/perovskite reactions are more composition-dependent. PEDOT:PSS acidity, NiO_x_ surface redox activity, fullerene morphology, and oxide surface hydroxyls can each influence the adjoining perovskite [62,63].

### 4.3. Defects and Trap-Assisted Degradation

Interfacial trap states are primarily caused by surface defects, chemical incompleteness, or non-ideal film formation processes, and they directly affect non-radiative recombination and carrier lifetime in the device [64]. At the perovskite/transport layer interface, common lattice misalignment, chemically unsaturated sites, or discontinuous regions can act as carrier trapping centers, increasing recombination losses and reducing the open-circuit voltage and short-circuit current. Particularly in perovskite films with rough surfaces or abundant pinholes, the trap-state energy levels formed at defect sites can induce local band distortion, impairing the efficiency of charge selective extraction [65].

If passivation is insufficient, deep-level traps may also form, serving as initiation sites for accelerated degradation. Therefore, high-quality interfacial control and passivation treatment have become key measures for improving stability.

## 5. Strategies for Improving Stability

### 5.1. Composition and Crystallization Engineering

Doping with different elements and at various ratios can modify the crystal structure of perovskites, achieving effects such as enhanced chemical bonding energy and strengthened lattice bonding (Figure 7a). This can effectively passivate defects, suppress the generation and migration of halide ions, and improve the intrinsic rigidity of perovskite materials. Meanwhile, high-quality crystallization of the perovskite film is critical for enhancing device performance and stability. By controlling the crystallization process and quality, and by steering the film toward a single preferred crystallographic orientation, the density of defect states in the film can be reduced and the carrier lifetime prolonged [66]. Dopants can act as crystallization modulators to optimize the phase composition and grain-boundary distribution of the film, thereby blocking pathways for moisture and oxygen ingress. The FA^+^ ion has a larger ionic radius but possesses higher inherent stability and lower volatility; thermodynamically, it is difficult to prepare high-quality films of the photoactive α-phase. Researchers have introduced MA^+^ ions to regulate the crystallization process of α-FAPbI_3_, enabling the fabrication of perovskite solar cells (PSCs) with high thermal stability and high efficiency. Similarly, many studies have focused on precise doping with elements such as Cs^+^ at the A-site and Br^−^ at the X-site to modulate the crystal structure and chemical bonding, thereby suppressing phase transitions and improving the stability of PSCs [44,45,67,68]. In MAPbI_3_, the hydrogen bond between the amine group and H–Br is stronger than that with H–I; therefore, appropriately substituting I^−^ with Br^−^ and partially replacing MA^+^ with FA^+^ can strengthen the interaction between the amine group/organic cation and the inorganic lattice, thereby enhancing perovskite stability [69,70]. The generation and migration of superoxide species in perovskites are key factors affecting oxygen stability. Consequently, many efforts have been devoted to suppressing superoxide formation to improve oxygen stability, such as focusing on A-site cation engineering to reduce the generation and migration of superoxide ions [71], ion-doping engineering of the ETL, and improving the perovskite/ETL interface to enhance the electron extraction rate and slow down superoxide generation, thereby achieving improved oxygen stability [72]. Iodine vacancies are the primary active centers for O_2_ reduction under illumination and dominate the formation of O_2_^−^ in perovskite films. Reducing iodine vacancies defects on the perovskite surface and in the bulk through surface passivation or ion-doping-mediated crystallization control represents another approach to suppress superoxide generation and diffusion, thus enhancing the photo-oxidative stability of the perovskite structure [9,73].

### 5.2. Defect Passivation

The defect concentration at perovskite interfaces is far greater than that in the bulk. Surface modification of perovskites—through approaches such as introducing organic molecular passivation to enhance coupling interactions, generating a two-dimensional perovskite layer at the interface of three-dimensional perovskites to passivate surface defects, and accelerating interfacial electron extraction—can not only achieve effective defect passivation but also prevent the introduction of heterogeneous structures into the interior of perovskite grains, reduce sensitivity to moisture and oxygen, minimize the impact of defects, and ultimately enhance the intrinsic stability of the perovskite. The mechanism of organic molecular passivation lies in using groups with lone-pair electrons, such as N, O, S, and P, as Lewis bases to form stable coordinate bonds with undercoordinated Pb^2+^ on the surface [76,77]. This coordination occupies highly active defect sites, blocking the starting points of water and oxygen adsorption and erosion at the source, while simultaneously plugging ion migration channels and releasing local lattice stress (Figure 7b). Certain molecules can also form a hydrophobic protective layer, synergistically enhancing stability at both the chemical and physical levels [10,78].

Numerous current studies focus on the passivation of perovskite defects by organic ammonium salts, whose core mechanism lies in the synergistic chemical passivation of both positive and negative charge defects on the film surface via the cationic and anionic components: the ammonium groups in the cations coordinate with iodide ions through hydrogen bonds to fill negatively charged defects such as halide vacancies, while the anions bind to undercoordinated Pb^2+^ through coordination bonds to passivate positively charged defects. Building on this bifunctional nature, researchers have further optimized the passivation efficacy through molecular structural engineering. On one hand, long-alkyl-chain cations tend to adsorb onto the perovskite surface in a parallel orientation, enhancing electrostatic interactions with defects; on the other hand, diammonium salts (PDADIs), possessing two ammonium groups, can simultaneously anchor multiple site defects, exhibiting stronger defect-blocking capability than monoammonium salts (PAIs). These chemical passivation effects ultimately significantly reduce the trap-state density of the films, suppress non-radiative recombination, prolong carrier lifetimes, and simultaneously optimize surface morphology and energy level alignment, leading to substantial improvements in both the power conversion efficiency and operational stability of inverted perovskite solar cells [79,80].

The use of zwitterionic molecules or multidentate ligands bearing oppositely charged or different coordinating groups enables synergistic defect passivation. For instance, in amino acids, the amino group (–NH_2_) can anchor to Pb^2+^ defects, while the carboxyl group (–COOH) interacts with halide vacancies or negatively charged defects through hydrogen bonds or ionic bonds. Such bidirectional anchoring can simultaneously passivate both positively and negatively charged defects, forming a stable multidentate chelating network. Some molecules can further evolve into an ultrathin polymer protective film through subsequent cross-linking reactions, enhancing mechanical and chemical stability [81]. Surface defect passivation engineering has evolved from simply eliminating non-radiative recombination into a comprehensive stability enhancement strategy that integrates chemical protection, structural stabilization, physical barrier formation, and strain regulation, representing a key technology for advancing perovskite solar cells toward industrialization. 

### 5.3. Dimensionality Engineering

Compared with three-dimensional perovskites, low-dimensional perovskites possess unique advantages in stability. Dimensionality engineering, through the construction of low-dimensional perovskite structures, provides an effective pathway to address the stability issues of perovskite materials. Considering the rules governing carrier transport behavior, two-dimensional perovskite structures have attracted the most intensive research attention. Researchers have attempted to introduce a 2D perovskite phase into 3D perovskite films to improve their stability (Figure 7c). 

If specific organic ammonium salts are chosen for passivation, a two-dimensional/three-dimensional perovskite heterojunction structure can be formed. Bulky organic ammonium salts react in situ with residual PbI_2_ on the perovskite surface to generate a thin, low-dimensional 2D perovskite capping layer. If the 2D layer contains hydrophobic long-chain organic cations as spacers, it can serve as a robust physical barrier that blocks external moisture penetration and the volatilization of internal organic cations, while effectively relaxing the residual tensile strain in the 3D perovskite to stabilize the photoactive phase. Simultaneously, the resulting band alignment can confine charge carriers within the 3D layer while passivating surface defects [82]. Based on the spatial configuration of the 3D and 2D perovskite components, such studies can be broadly categorized into 2D/3D mixed-phase perovskites and 2D/3D heterojunction perovskites. 2D/3D mixed-phase perovskites refer to mixed-dimensional structures in which 2D perovskites with different *n* values and 3D perovskites coexist within a single film. This internal multi-phase heterostructure forms a series of coordinated phase-domain interfaces that effectively promote the relaxation of interfacial strain. Coherent growth between phase regions with different *n* values helps to accommodate lattice mismatch, thereby suppressing the formation of structural defects and thermal decomposition centers.

Moreover, the high formation energy and inherent chemical stability of the low-dimensional layers provide a robust scaffold that reinforces the crystalline framework of the 3D perovskite. This structural reinforcement not only enhances the resistance of the film to thermally induced phase degradation but also ensures a more durable and high-quality photoactive layer [83], which is critical for the sustained performance of the resulting devices. However, the quantum well effect introduced by the 2D mixed phases, the obstruction of charge transport by the spacer cations in the 2D perovskite, and the damage to the 3D perovskite during the formation of the 2D phase can potentially compromise the efficiency and stability of PSCs. 

### 5.4. Crystal-Facet and Orientation Engineering

The differences in atomic arrangement and chemical environment exposed on different crystal facets are key to defect formation and the binding strength of passivating molecules, leading to intrinsic differences in the tolerance of each facet to water molecules, oxygen molecules, electric field response, and thermal stress. Consequently, crystal facet engineering has become a research focus in recent years (Figure 7d). The (111) facet possesses a significantly higher areal packing density than the (100) facet, and therefore exhibits outstanding stability, as has been repeatedly confirmed in multiple independent studies [84,85,86,87,88], DFT calculations and humidity exposure experiments have demonstrated that the adsorption energy of water molecules on the (100) facet is far greater than that on the (111) facet, and that –OH species form more readily on the (100) facet, triggering the α→δ phase transition. Moreover, the dense atomic arrangement and higher ion migration activation energy of the (111) facet significantly suppress the migration of I^−^ and FA^+^. Although the (110) facet is more prone to forming defects, these are all shallow-level defects that do not act as non-radiative recombination centers. To maximize the exposure of (111) facets in the film, a bidentate nitrogen-based chelating ligand, TMePhen, has been employed. It forms a five-membered chelate ring with Pb^2+^ through its two N atoms and templates the growth of the (111) facet; its molecular configuration is highly similar to the atomic arrangement of the (111) facet, achieving a configuration-matching effect, ultimately retaining 92% of the initial efficiency after 840 h of room-temperature storage [84]. Using natural tea saponin (TS) to modify SnO_2_, the coordination of C=O with Pb^2+^ and the hydrogen bonding of –OH with FA^+^/I^−^ guide the perovskite to grow with a (111) orientation starting from the buried interface, achieving 90% initial efficiency retention after 800 h of storage at 20% RH [87]. In the SACR strategy, CHA reacts with FA^+^ to generate the CHAFA^+^ bulky cation, which acts as a strong capping agent to suppress (100) facet growth and indirectly purify the (111) orientation. Devices with (111)-oriented facets retained >95% of the initial efficiency after 2000 h, representing some of the best stability data reported to date [85]. The seed crystal induction method, in which seed crystals with specific crystal facets are pre-embedded so that subsequent crystallization follows the orientation of the seeds, sets the crystallographic orientation at the onset of crystallization. This method is a mature process in the conventional semiconductor industry but is still in its infancy for perovskites, despite its enormous potential [89]. However, not all (111) facets are inherently stable. In FAPbI_3_, the (111) facet exhibits a high defect density and severe non-radiative recombination, and its stability advantages may be offset by defect-induced degradation in actual devices [90]. The stability of the (111) facet only manifests under the precondition of low defect density. If the (111) facet itself is riddled with deep-level defects, these defect sites still serve as entry points for moisture ingress and ion migration; thus, crystal facet engineering must be advanced synergistically with defect passivation.

### 5.5. Strain and Thermomechanical Engineering

Among the various degradation mechanisms, structural instability triggered by thermomechanical stress is increasingly recognized as a fundamental scientific issue that spans the material, interface, and device architecture. In recent years, strain engineering, centered on stress regulation, has evolved from passive failure analysis to active design, becoming a bridge linking efficiency and stability. The cumulative time of thermal exposure has a greater impact on performance degradation than the number of thermal cycles, revealing the irreversible accumulation characteristic of thermal stress damage. Stress is not only a driving force for physical failure but also accelerates chemical degradation and electrical decay. Early strategies focused on reinforcing the weak regions where stress concentrates the most, emphasizing enhanced resistance at interfaces and grain boundaries. By introducing an in situ polymerized network of α-lipoic acid at perovskite grain boundaries and incorporating a sulfonium-based derivative, DMSLA, into the self-assembled monolayer of the hole transport layer, a dual enhancement of grain boundary cohesion and interfacial adhesion was achieved. This approach, by increasing the cohesive fracture energy of the material, enables the device to withstand greater thermomechanical loads without delamination or cracking [91]. However, such strategies still belong to passive stress accommodation, with their tolerance limits constrained by the intrinsic strength of the materials. A more ingenious strategy is to endow grain boundaries with dynamic responsiveness. A molecular complex that remains in a fluid state over a wide temperature range was creatively designed (Figure 8a). After spontaneously enriching at the grain boundaries, it fully accommodates the expansion and contraction of the perovskite lattice through its own flow and deformation during temperature fluctuations, fundamentally eliminating stress concentration at rigid grain boundaries [92]. This fluid buffering mechanism represents a paradigm shift in stress regulation from static defense to dynamic adaptation, with effects manifested in a reduction of lattice strain amplitude by over 40% and the near-complete recovery of optoelectronic performance after thermal cycling. Addressing the issue from the thermal source, highly thermally conductive ortho-Carborane molecules were introduced into Sn–Pb mixed perovskites, increasing the film thermal conductivity by approximately 9% and reducing the device surface operating temperature by about 5 °C [93]. Although this thermal management strategy does not act directly on stress, it fundamentally reduces the driving force for thermal stress by diminishing the amplitude of temperature fluctuations and suppressing the formation of localized hot spots. This is particularly critical for Sn–Pb systems, which inherently possess inferior thermal stability. By introducing a bifunctional molecule, PYA, during the intermediate state of the annealing process and combining it with UV crosslinking, a phase-pure 2D perovskite nanonet was simultaneously constructed on the top surface, along the grain boundary network, and at the buried interface of the perovskite. This crosslinked network serves not only as a chemical passivation layer but also as a mechanically rigid skeleton that omnidirectionally encapsulates the 3D grains. Through mechanical confinement, it effectively suppresses lattice deformation induced by the electrostrictive effect and raises the threshold for high-field-driven ion migration by a factor of 2.3 to 4.8 [94]. This active locking mechanism directly converts mechanical reinforcement into chemical/electrical suppression of ion migration and phase transitions, enabling the device to retain over 95% of its initial efficiency after 2400 h of thermal storage at 85 °C and achieve an efficiency retention rate of 97% after 500 thermal cycles. Strain engineering has thus evolved from the passive modification of individual interfaces into a multi-dimensional systematic design encompassing thermal regulation, mechanical buffering, and chemical locking.

Employing elastic polymers, ionic liquids, or low-dimensional materials as a buffer layer on the cathode side of the device can effectively absorb stress arising from thermal expansion and contraction, thereby alleviating interfacial delamination (Figure 8b). At the same time, such a flexible buffer layer provides a physical barrier for the perovskite layer, retarding the diffusion of moisture and oxygen, and enhancing mechanical stability and long-term encapsulation performance.

### 5.6. Encapsulation and Internal Barrier Design 

Encapsulation serves as the last line of defense when moving laboratory devices into real-world environments. Without effective encapsulation, even the most superior internal materials cannot easily withstand the attack of external moisture, oxygen, and light (Figure 8c). Therefore, the design of an encapsulation strategy must not only demand excellent barrier properties from the materials but also take into account flexibility, transparency, and process compatibility [97]. PDMS encapsulation significantly suppresses moisture and oxygen erosion of the perovskite layer. XRD results show that the PbI_2_ peak weakens after encapsulation, indicating that PDMS enhances the intrinsic stability of the perovskite through passivation of Pb^0^ defects and its hydrophobic nature [98]. To address the degradation issue, an encapsulation strategy is proposed that employs a hydrophobic phenethylammonium iodide (PEAI) coating as a barrier against moisture and oxygen in the environment. This approach effectively stabilizes the perovskite material, preserving its efficiency even upon exposure to water and air [40]. Glass encapsulation combined with epoxy edge sealing is currently the most common packaging approach for rigid devices, offering good optical transparency and gas-barrier capability. UV-curable resins are also widely used in flexible encapsulation, but their sealing performance against moisture remains limited; therefore, they need to be used in conjunction with other encapsulation layers [99].

New encapsulation strategies are trending toward multifunctional layered structural designs. For example, a superhydrophobic organic layer is applied as an inner coating to prevent moisture adsorption, an inorganic barrier layer is then introduced to enhance the resistance to water vapor transmission (WVTR), and the outermost layer employs flexible polymers like PET or PI to provide mechanical protection. This sandwich-like encapsulation architecture can significantly retard the degradation rate of perovskite, extending device operational lifetime from hundreds of hours to thousands or even tens of thousands of hours [99]. To address the issue of internal ion migration in perovskite solar cells (PSCs), Li et al. developed an internal encapsulation strategy based on PTFE. This ultrathin layer, placed at the interface between the perovskite and the electron transport layer, not only effectively blocks moisture but, more importantly, significantly suppresses ion migration, thereby simultaneously improving the device’s efficiency and intrinsic stability. The PTFE-encapsulated device achieved a power conversion efficiency of 25.49% and retained 92.6% of its initial efficiency after 1500 h of aging, far outperforming the control group. This work provides an effective interfacial engineering solution to the long-term stability challenge of PSCs [100].

### 5.7. Stable and Lower-Toxicity Material Systems

The structural properties of the perovskite material system itself are the fundamental factor governing its stability. Therefore, starting from the material source and developing novel materials with high stability and low toxicity has become an important direction for enhancing the sustainability of perovskite solar cells (Table 1).

Substituting Pb^2+^ with Sn^2+^ is the most direct route to lead-free perovskites. However, Sn^2+^ is highly susceptible to oxidation to Sn^4+^ in air, leading to the collapse of the perovskite structure (Figure 8d). To suppress this process, researchers have proposed utilizing the synergistic effect of AHP doping and the CuSCN hole-transporting material to improve film morphology and reduce defect density [105]. Nevertheless, the trade-off between efficiency and stability still needs to be addressed, narrow-bandgap all-inorganic perovskites based on tin–lead (Sn–Pb) systems still face challenges such as poor crystallization quality, facile oxidation of Sn^2+^, and high defect density.

Bi-based perovskite solar cells are a low-toxicity, lead-free family in which Bi^3+^ substitutes for Pb^2+^. Most members fall into two groups: A_3_Bi_2_I_9_ type low-dimensional bismuth halides and A_2_M^+^Bi^3+^X_6_ type double perovskites. Their main attraction is reduced toxicity together with good tolerance to moisture and heat in air. The structural limitation comes from the trivalent nature of Bi^3+^, it does not readily support an ideal three-dimensional ABX_3_ framework, favoring instead 0D/2D low-dimensional structures or double perovskites [106,107]. These phases typically exhibit wide, often indirect band gaps, strong exciton binding, and limited carrier mobility. As a result, single-junction PCE generally remain around 3–3.5% or lower, well below those of Pb-based devices. Double-perovskite solar cells did draw attention by reaching 8% PCE, but the family as a whole still struggles to compete with Pb- and Sn-based systems. Overall, Bi-based perovskites currently represent a stability-first but efficiency-limited route [108]. They are more compelling as top absorbers in tandem cells, UV photodetectors, or stable optoelectronic device candidates than as a direct replacement for high-efficiency single-junction solar cell.

Ge-based perovskites have attracted attention as lead-free alternatives, largely because Ge^2+^ resembles Pb^2+^ in valence state, ionic size, and electronic configuration. MAGeI_3_ and FAGeI_3_ exhibit relatively wide bandgaps, while CsGeI_3_ has a bandgap near 1.6 eV closer to the optimum for single-junction cells. Their promise is tempered, by practical constraints, Ge^2+^ is prone to oxidation to Ge^4+^, crystallization is challenging, films tend to be defect-rich, interfacial recombination is severe, and Ge is both costly and scarce [109]. As a result, pure Ge-based perovskite solar cells have generally delivered modest experimental efficiencies at relatively high cost, making them a less attractive absorber layer at this stage.

### 5.8. Section Summary

The most reliable stability strategies act on more than one elementary process while avoiding a new transport penalty. Composition engineering can stabilize a phase but alter the bandgap; 2D layers can block reactants but impede carriers; stronger lattices can transfer stress to interfaces; and added interlayers can create new chemical contacts. Strategy evaluation should therefore report initial efficiency, stabilized output, failure mode, stress conditions, encapsulation status, and whether the proposed mechanism remains valid during aging.

## 6. Conclusions

To enable inverted perovskite solar cells (PSCs) to truly move from the laboratory to application, future efforts should not focus solely on a single material. Instead, joint progress is needed in intrinsically stable materials, process–encapsulation co-optimization, unified testing standards, and cross-disciplinary assessment. Methodologically, density functional theory (DFT) and molecular dynamics (MD) can be linked with device physics; ion migration, phase segregation, and defect reactions can be incorporated into degradation kinetic equations, and lifetime research can be shifted from empirical description to quantitative prediction [110]. High-throughput computing and automated experiments can then be combined with machine learning (ML) to actively screen the composition/interface space, while data are consolidated into a “composition–process–stress–lifetime” database, moving research from passive observation to proactive prognostics. On the experimental side, in situ X-ray photoelectron spectroscopy (XPS), photoluminescence (PL) imaging, grazing incidence wide-angle X-ray scattering (GIWAXS), and electrical measurements should be integrated synchronously to track the spatiotemporal evolution of chemical states, crystalline phases, defects, and the built-in electric field, so as to validate models and reveal failure pathways. Outdoor arrays and multi-stress coupled aging platforms can distill real-world scenario data into accelerated lifetime testing protocols and build a lifetime assessment system that is closer to practical application. These routes are not simply parallel but interlocking: kinetic models provide physical support for ML, high-throughput and in situ characterization provide data and validation, and long-term scenario testing in turn calibrates theory and predictions.

Graph Neural networks (GNNs) can integrate molecular and perovskite device-level features to directly predict the final device power conversion efficiency PCE in an end-to-end manner, thereby enabling low-cost, high-throughput screening. Moreover, self-attention mechanisms can provide atom- and bond-level importance to enhance interpretability, accelerating materials discovery with final device performance as the direct optimization objective [110,111,112].

An AI closed-loop research and development framework, driven by machine learning for materials discovery and automated manufacturing and optimization, can accelerate the entire workflow from molecular discovery to high-reproducibility device fabrication. On the materials side, AI combines DFT, SISSO symbolic regression, SHAP analysis, and NGBoost active learning to rapidly screen interface passivation materials from candidate molecules, leading to the discovery and validation of highly efficient passivation molecules. On the manufacturing side, AI uses automated platforms and Bayesian optimization for closed-loop iterative optimization of perovskite layer and passivation layer processes, achieving better reproducibility than manually fabricated perovskite solar cell devices. This is a replicable and generalizable independent R&D pathway that is highly likely to transform how materials and devices are researched and manufactured in the future [113].

This review presents a systematic overview of the stability issues in inverted perovskite solar cells, covering aspects including structural design; the influence of moisture, heat, light, oxygen, interfacial reactions and ion migration mechanisms; and strategies for stability enhancement.

## Figures and Tables

**Figure 1:**
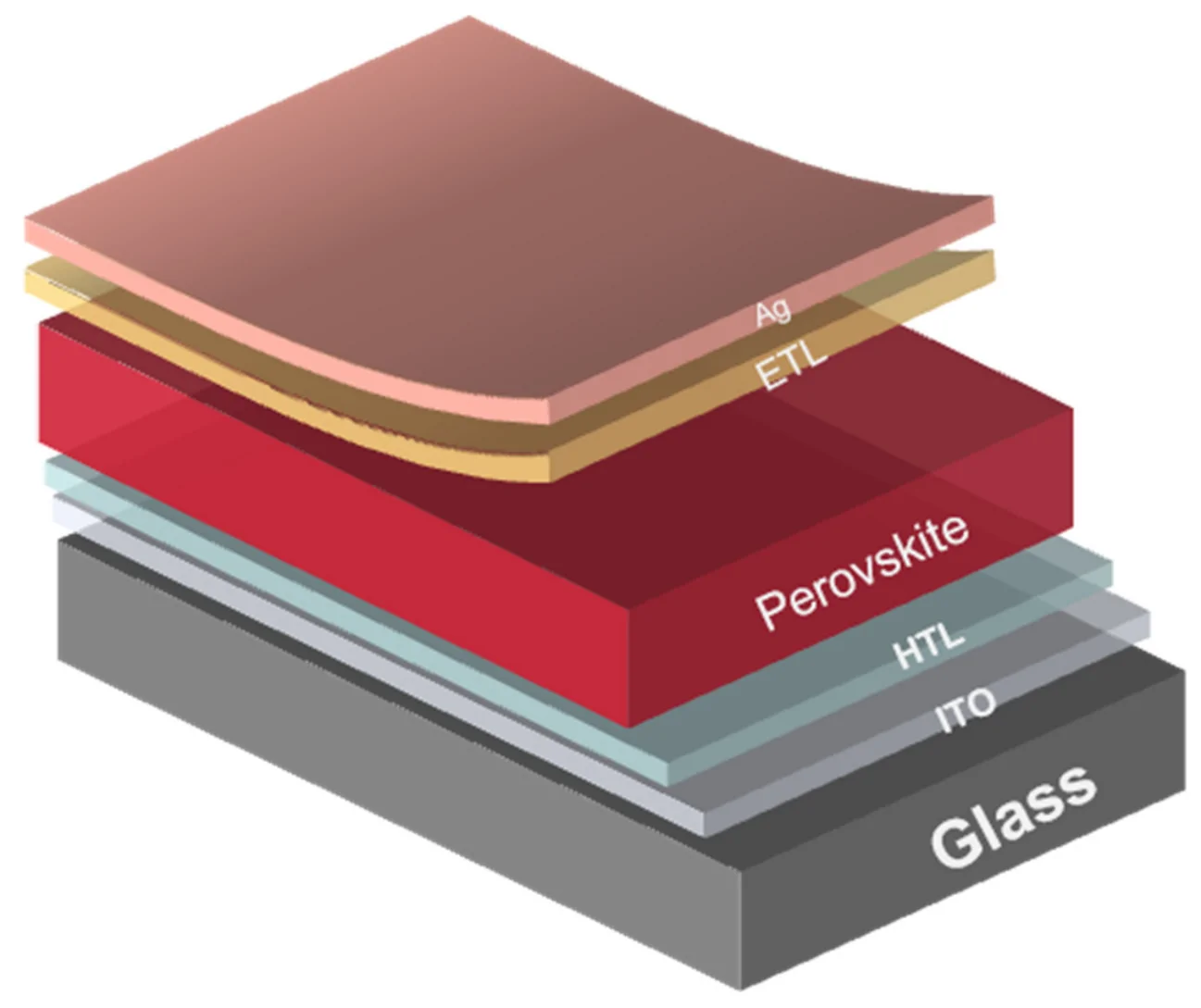
Inverted p-i-n structured perovskite solar cells.

**Figure 2:**
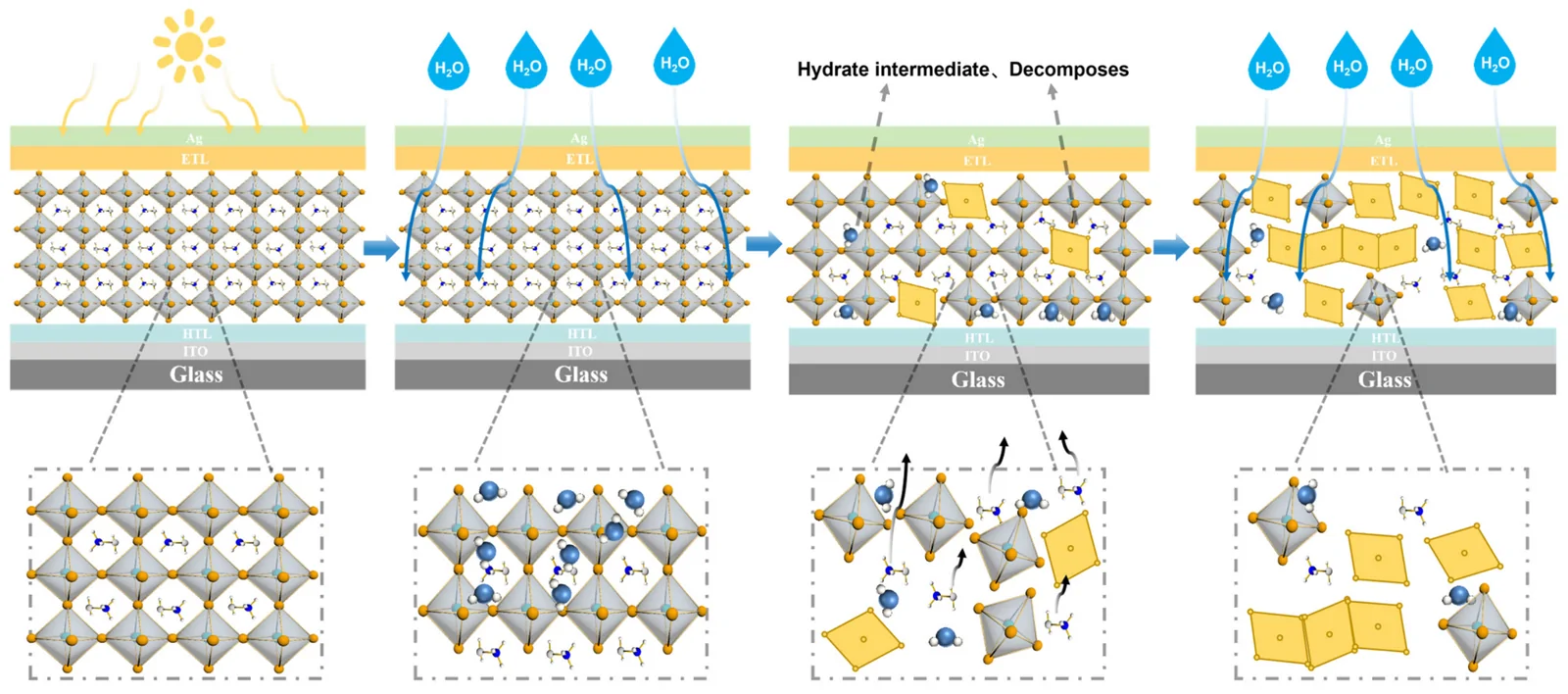
Moisture-ingress-induced degradation pathways in perovskite solar cells: Water molecules initially adsorb preferentially at iodine vacancies, undercoordinated Pb^2+^ sites and grain boundaries, where they hydrogen-bond to halides and coordinate with Pb^2+^. Continuous water ingress then drives lattice evolution, forming hydrated intermediates that decompose, causing framework collapse and byproduct release. Ultimately, the perovskite structure is severely destroyed with abundant PbI_2_ formation, leading to device failure.

**Figure 3:**
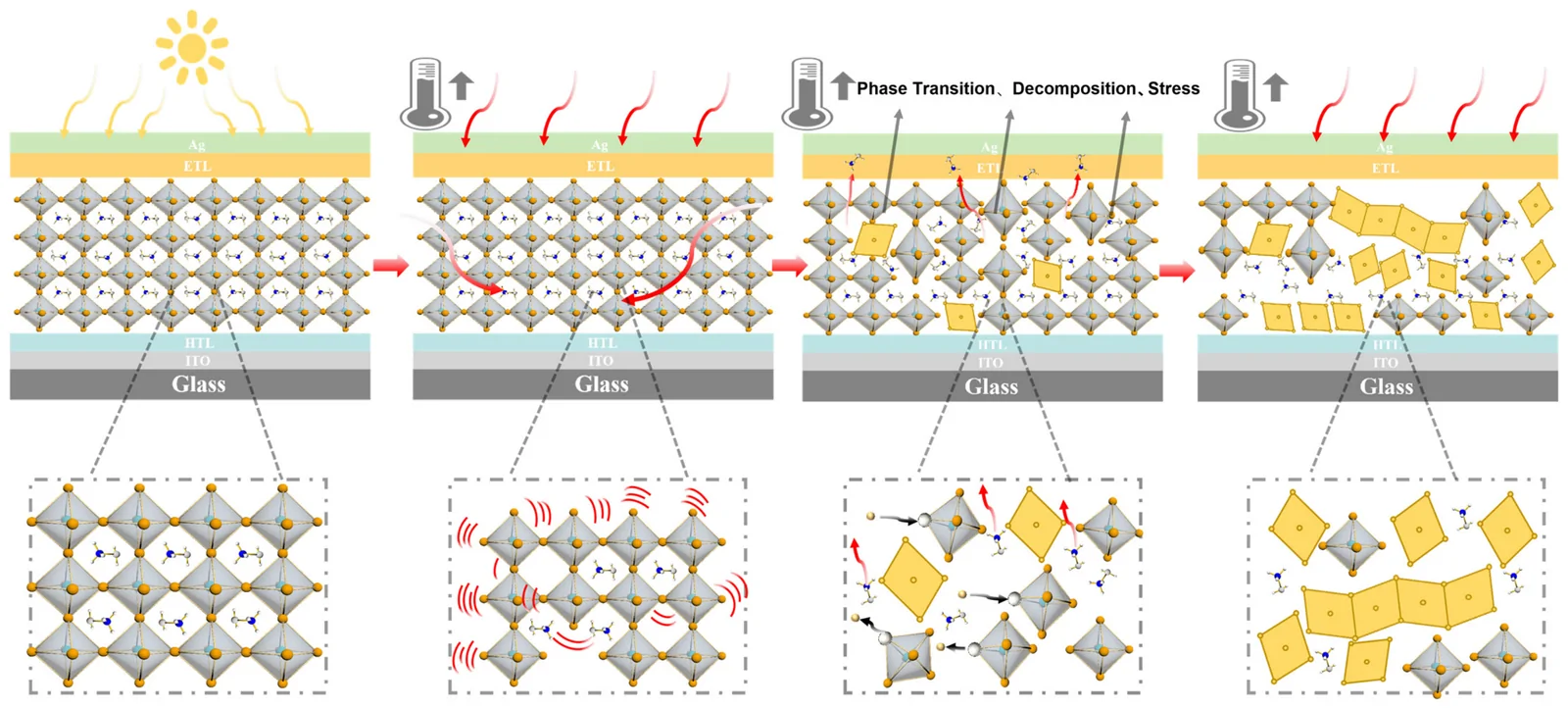
Thermal degradation mechanisms and pathways of perovskite solar cells: In the initial stage, heat accumulates within the perovskite lattice and initiates ion migration. As heat continues to accumulate, ion migration intensifies and induces lattice distortion, causing the material to undergo a phase transition and decompose. Ultimately, the lattice structure collapses completely, resulting in extensive decomposition into yellow PbI_2_.

**Figure 4:**
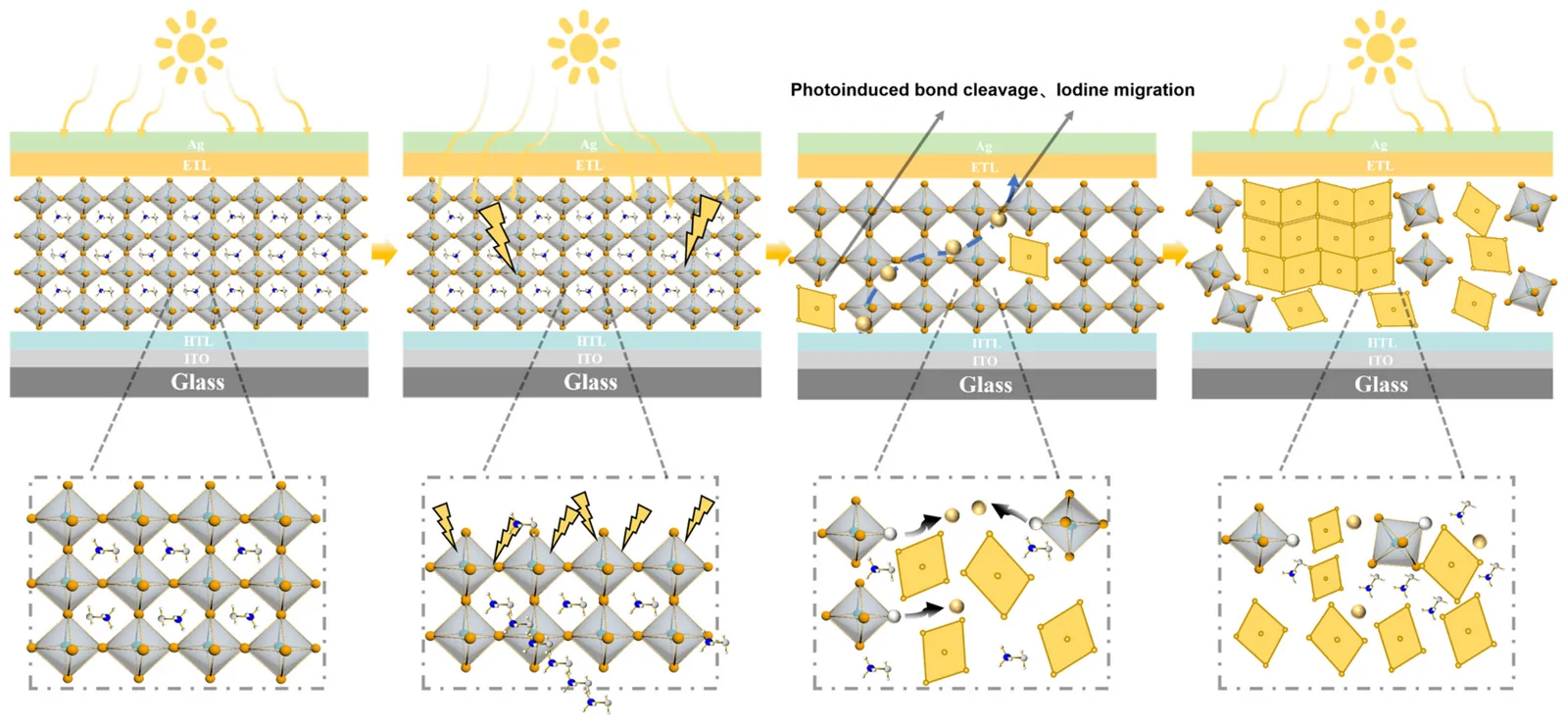
Light degradation mechanisms and pathways of perovskite solar cells: Under combined light and heat, the initial stage involves light-induced bond cleavage and heat-driven ion migration, causing lattice distortion and triggering a phase transition. Concurrent iodine loss and electron-induced reduction of Pb^2+^ to Pb form iodine-poor, lead-rich regions that catalyze further decomposition. Ultimately, the lattice collapses, and the material decomposes largely into yellow Pb^2+^ and other products.

**Figure 5:**
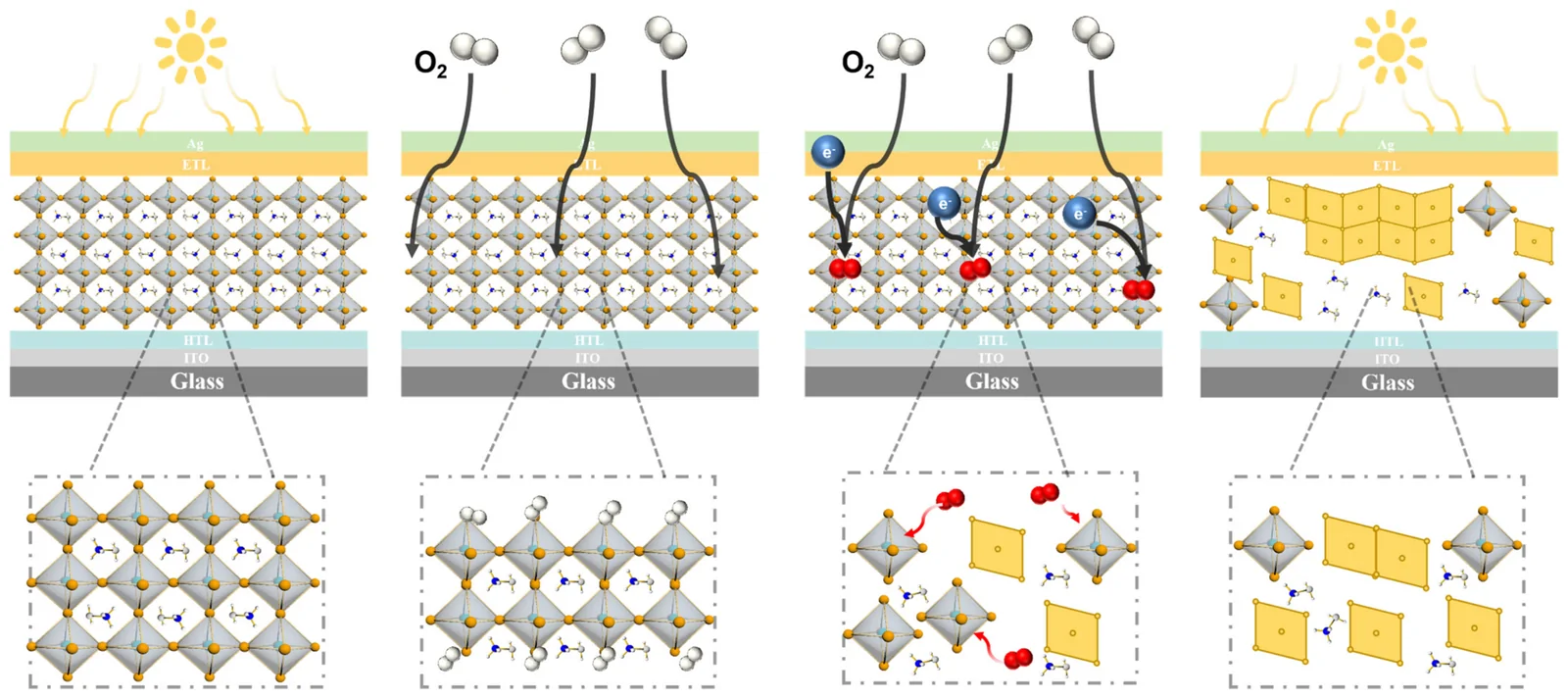
Oxygen degradation pathways of perovskite solar cells: Upon illumination, O_2_ molecules initially adsorb at defect sites on the perovskite surface. Photogenerated electrons then transfer from the perovskite conduction band or defect states to O_2_, generating highly reactive superoxide anions O_2_ that rapidly initiate irreversible decomposition of the material. Eventually, the lattice collapses completely, and the material undergoes extensive decomposition to form yellow PbI_2_.

**Figure 6:**
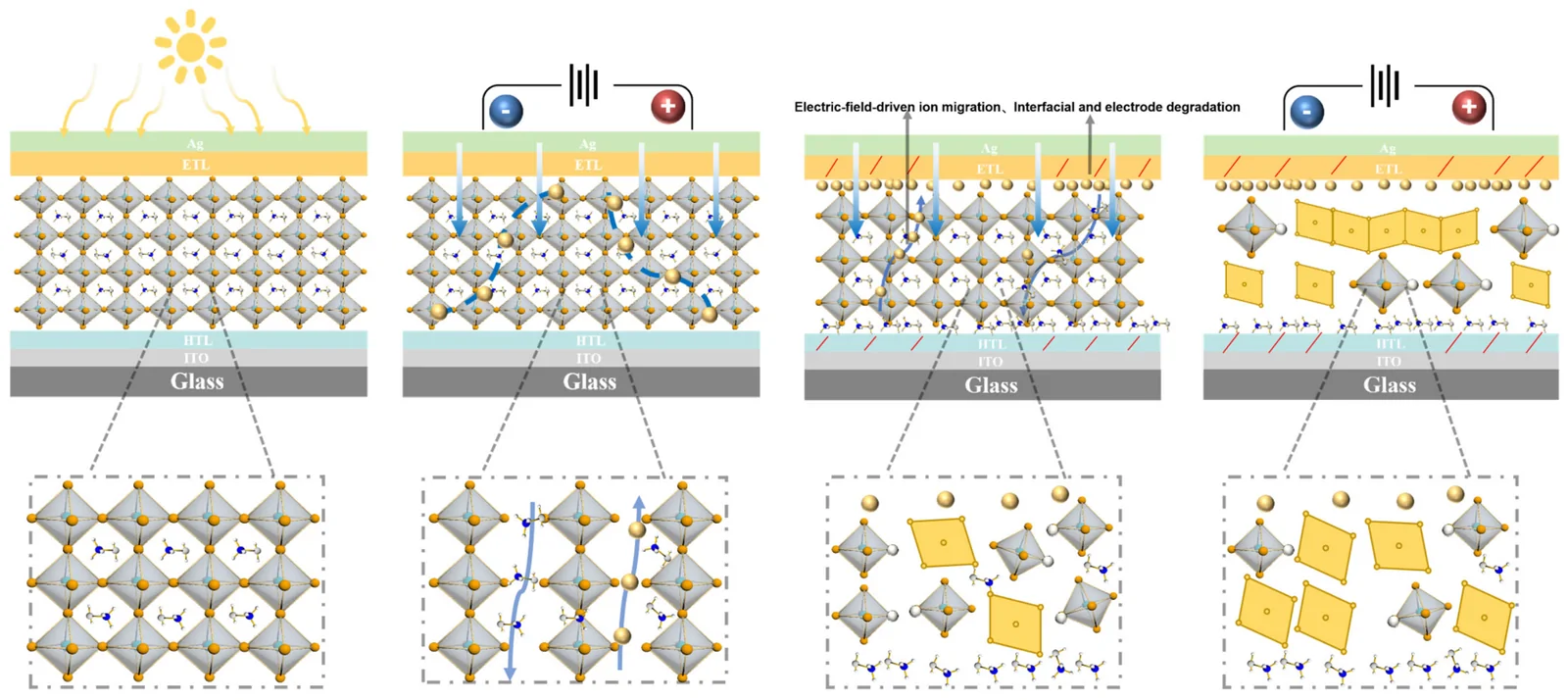
Electric-field-induced degradation pathways of perovskite solar cells: Under an applied external bias, cations and anions are driven by the electric field to migrate toward the opposing interfaces, leaving vacancies behind and thereby inducing lattice distortion and collapse. With continued bias, ions accumulate extensively at these interfaces, triggering irreversible chemical reactions.

**Figure 7:**
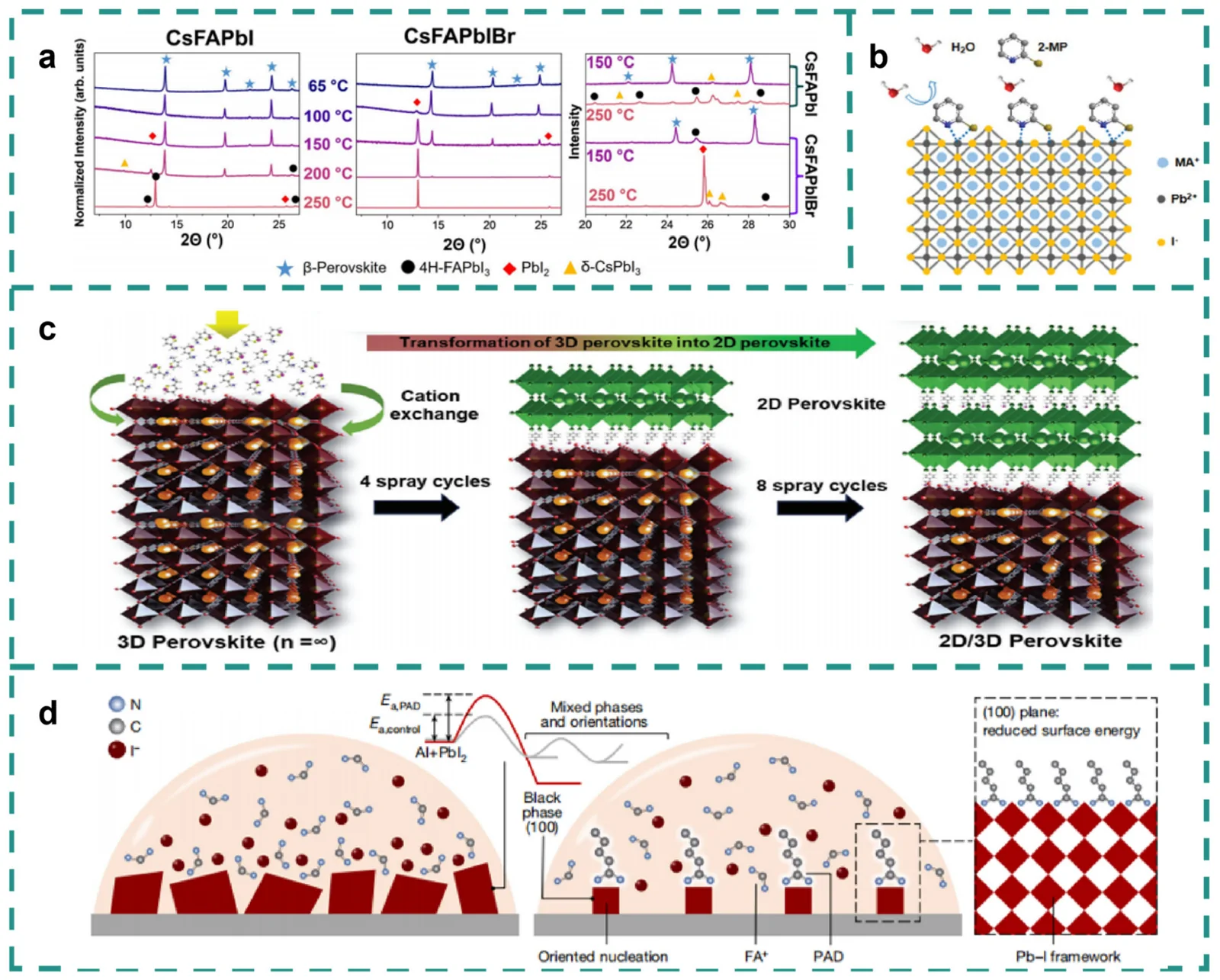
(**a**) Zoom-in on high angles of XRD patterns of CsFAPbI and CsFAPbIBr at 150 and 250 °C; [67]; (**b**) The mechanism for 2-MP passivated surface preventing the water from the perovskite surface [74]; (**c**) Transformation of 3D perovskite into 2D perovskite [75]; (**d**) Schematic illustration of the thermodynamic driving force and the kinetics of the oriented nucleation of perovskite films [46].

**Figure 8:**
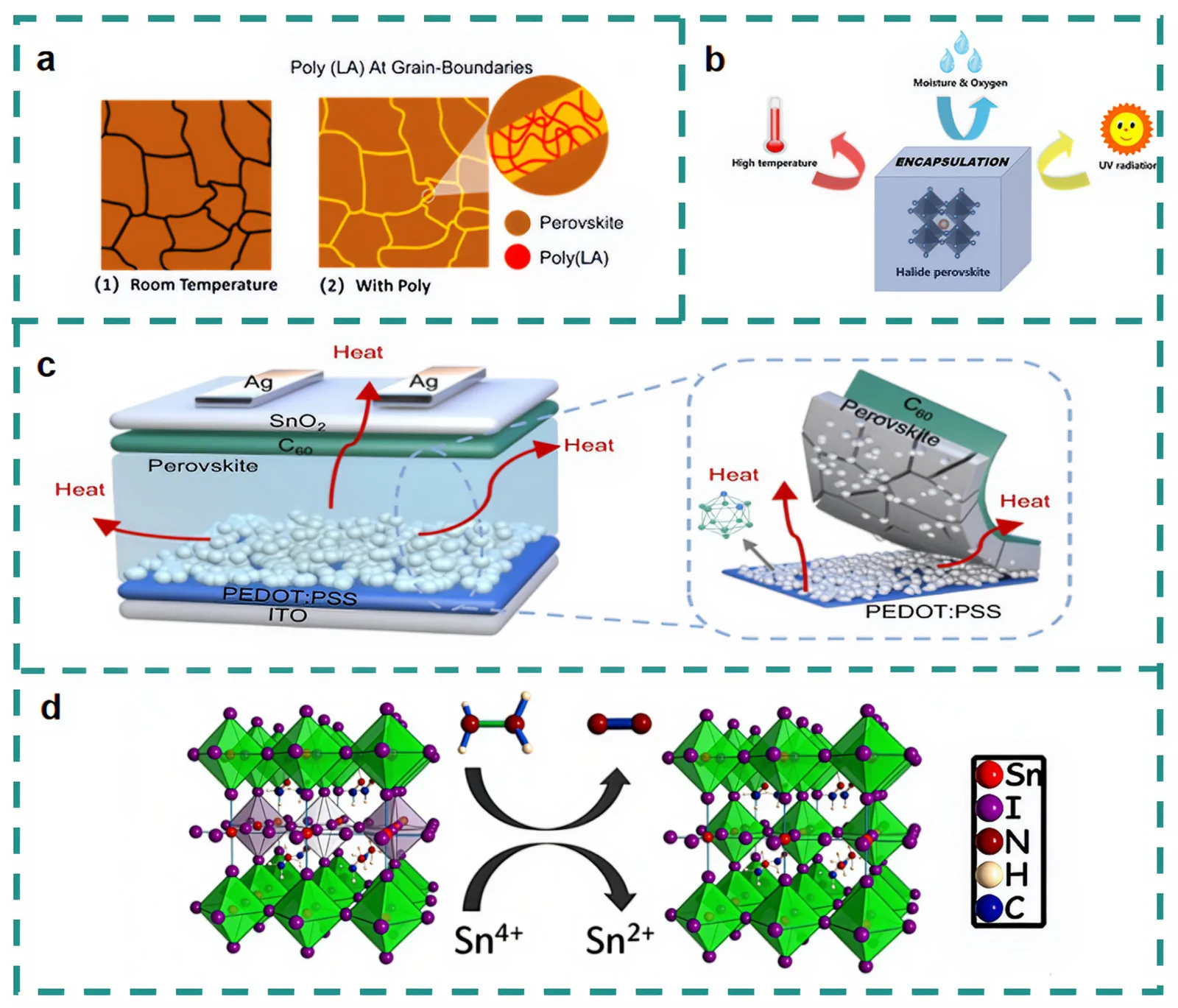
(**a**) Perovskite polycrystalline structures with and without poly (LA) at the grain boundaries [91]; (**b**) Schematic illustration of protecting perovskite materials from environmental stress through encapsulation [95]; (**c**) Configuration of the p-i-n device treated with o-CB and schematic diagram of the heat dissipation [93]; (**d**) Tin-based halide perovskite materials [96].

**Table 1:** Advantages and disadvantages of different perovskite material systems.

Base	System	Advantage	Disadvantage	Reference
Pb	APbX_3_	PCE Cost	Toxicity Stability	[101]
Sn	ASnX_3_	Toxicity Bandgap	Oxidation Defect density	[102]
Bi	A_3_Bi_2_I_9_ A_2_M^+^Bi^3+^X_6_	Toxicity Stability	Film quality PCE	[103]
Ge	MAGeI CsGeI_3_	Elemental abundance	Stability Film quality	[104]

## Data Availability

No new data were created or analyzed in this study. Data sharing is not applicable to this article.
